# Supplementary material for: A new juvenile sauropod specimen from the Middle Jurassic Dongdaqiao Formation of East Tibet
Source: PeerJ. 2023 Mar 22;11:e14982. doi: 10.7717/peerj.14982 (PMC10039653; doi:10.7717/peerj.14982)
Supplement: Supplemental Information 1 [file peerj-11-14982-s001.pdf]

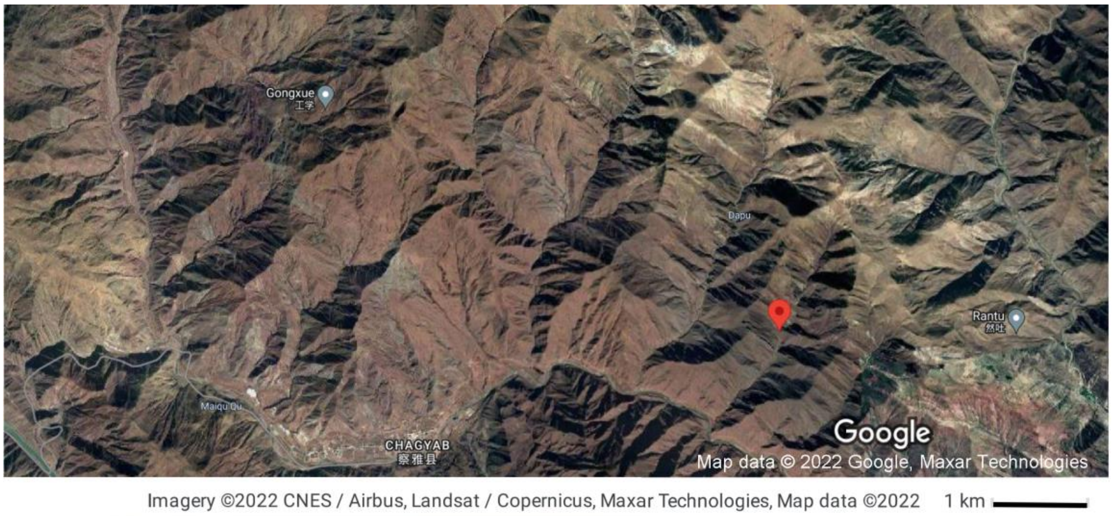

Fig. S1. Satellite image showing the fossil site that yielded the sauropod material described in this paper (CGS V001) in Chaya County, Qamdu District, East Tibet. Map data © 2022 Google, Maxar Technologies.

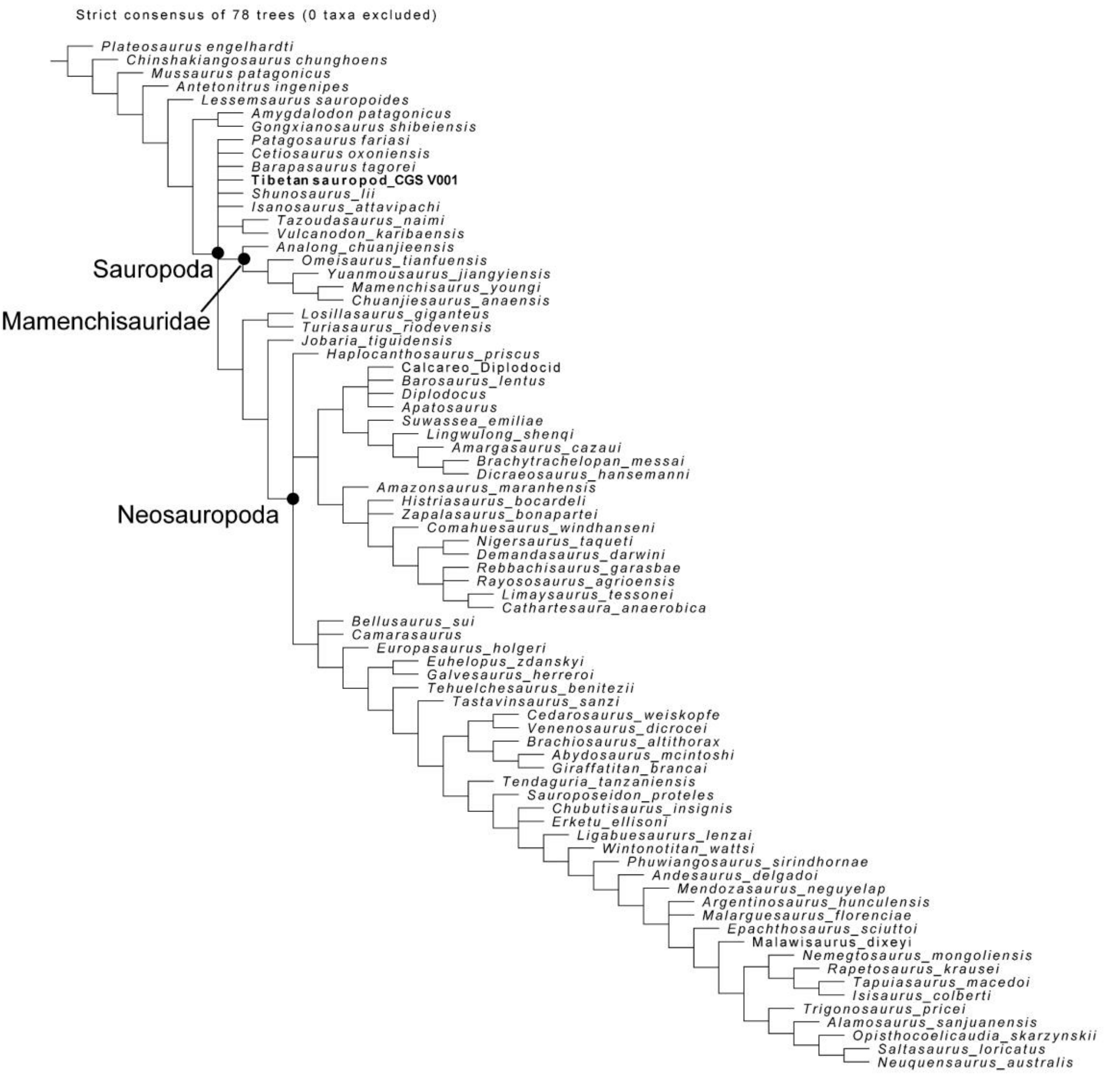

Fig. S2. Strict consensus tree from 78 MPTs (TL=1223) based on 77 taxa and 386 characters. Data matrix modified from Ren et al. (2021).
